# Blockchain-based shop floor control system for small and medium-sized enterprise evolution to industry 4.0

**DOI:** 10.1038/s41598-025-13809-3

**Published:** 2025-08-05

**Authors:** Chin-Te Lin

**Affiliations:** Department of Mechanical Engineering, National Central University, No. 300, Zhongda Rd., Zhongli District, Taoyuan City, 320317 Taiwan, ROC

**Keywords:** Small and medium-sized enterprises, Blockchain, Hyperledger fabric, Smart contract, Shop floor management, Industry 4.0, Digital thread, Mechanical engineering, Information technology, Software

## Abstract

Small and medium-sized enterprises (SMEs) require agile and robust strategies to navigate the difficulties of their evolution toward Industry 4.0. This study proposes a Blockchain-based Shop Floor Control System (BSFCS) that incorporates a Consensus-based Workflow Change Integration (CWCI) mechanism and Smart Contract Service (SCS) design methodology to provide these strategies. The CWCI represents an incremental agile approach, enabling real-time workflow adjustments and smooth, rapid integration of evolving enterprise requirements. The modular architecture and optimized key-state data structures that are features of the SCS design methodology support efficient transaction management, ensuring system feasibility. A prototype implementing the BSFCS was tested on a realistic industrial platform replicating real-world manufacturing conditions. Results from two validation experiments demonstrate significant performance improvements over a standard model. These enhancements include dynamic workflow adaptability, enhanced operational transparency, and efficient handling of real-time changes. These BSFCS characteristics represent requirements SMEs need for a reliable and sequential pathway they can follow implementing Industry 4.0. They show that compared to existing frameworks, the BSFCS framework we propose offers superior adaptability and scalability. It particularly excels in handling dynamic workflow changes and sequential upgrades with minimal disruption to ongoing operations.

**Supplementary Information:**

The online version contains supplementary material available at 10.1038/s41598-025-13809-3.

## Introduction

Small and medium-sized enterprises (SMEs) play a critical role in the global economy, particularly in manufacturing. In Taiwan, SMEs represent 98.9% of all companies, with manufacturing SMEs contributing 8.88% to this total. These businesses employ 20.4% of the workforce and account for exports valued at $452.74 billion^1^. SMEs are recognized for their adaptability, innovation, and ability to deliver diverse, customized, and high-quality products at competitive prices^2,3^. Their flexibility, entrepreneurial spirit, and close customer interaction are features that distinguish them from larger enterprises^3,4^.

Despite these strengths, transitioning toward Industry 4.0 (I4.0) presents significant challenges for SMEs. While I4.0 technologies—such as the Internet of Things (IoT), cloud computing, and machine learning—offer opportunities for improving production efficiency, decision-making, and supply chain integration, SMEs often struggle with operational inertia, limited resources, and unsatisfactory technological frameworks, hindering their adoption of these systems^2,5^.

To overcome these challenges, SMEs require solutions that are incremental and agile to minimize disruption to existing operations while allowing continuous adaptation to evolving production demands^2^. Recent research highlights the transformative potential of blockchain technology (BCT) to meet these demands. The survey of Alazab and Alhyari^6^ identifies key BCT benefits that include enhanced data security, product traceability, and efficient resource utilization. These features align closely with the objectives of Industry 4.0, where real-time data sharing and automated processes are critical for achieving operational excellence and sustainability. By leveraging BCT, this study proposes an agile and incremental deployment approach that does ensure gradual system upgrades while maintaining operational stability. The proposed strategies in this study include: (1) Dynamic Smart Contract Development: Continuously updating smart contracts to reflect changes in industrial processes, ensuring automation efficiency and system flexibility. (2) Scalability Solutions for Blockchain: Adopting techniques, like sharding and partial replica storage, to optimize blockchain performance while maintaining data integrity^7–9^.

BCT is a promising framework that could address the challenges faced by SMEs, particularly their flexibility and scalability issues. Supporting agile methodologies, this study proposes an incremental deployment strategy through a Blockchain-based Shop Floor Control System (BSFCS) framework that integrates Hyperledger Fabric (HLF) and smart contracts to provide real-time production control, dynamic workflow adaptability, and transparent data management. The core innovation of this study lies in a Consensus-based Workflow Change Integration (CWCI) mechanism that facilitates incremental digital transformation by dynamically adapting production workflows to real-time changes.

This study makes the following contributions. First, it introduces a CWCI mechanism that combines agile methodologies with blockchain-based smart contracts to enable real-time workflow adjustments and continuous system upgrades. CWCI facilitates the rapid, problem-free digitization of workflow consensus on required workflow changes, ensuring SMEs can adapt dynamically to evolving production demands and achieve incremental digital transformation with minimal disruption. Second, it develops a BSFCS architecture designed to address the scalability and adaptability challenges of changing the shop floor environment. Featuring a modular design, smart contract services, and optimized key-state data structures, the BSFCS enables efficient data synchronization and decentralized communication, providing SMEs with a robust and scalable foundation for Industry 4.0 upgrades. Lastly, the study validates the system through practical implementation in an experimental factory according to real-world manufacturing conditions. The two-phase validation—including initial deployment and dynamic workflow adjustments through engineering change orders (ECOs)—demonstrates the system’s feasibility, adaptability, and practical value in addressing the real-time production challenges of SMEs.

The remainder of this paper is organized as follows. “Literature review” reviews the related literature and identifies research gaps. “Methodology” details the design methodology and architecture of BSFCS. “System implementation” describes the implementation process and experimental setup. “Testing” presents testing cases and performance results. “Discussion and limitations” discusses significant findings and key challenges. “Conclusion” concludes the study and suggests future research directions.

## Literature review

### SME challenges and needs

Small and medium-sized enterprises (SMEs) face unique challenges in their evolution toward Industry 4.0. Rauch et al.^10^ identified functional requirements for SMEs using axiomatic design, providing insights into key manufacturing system goals. The major challenges and corresponding needs are outlined below:

- **Digitalized network**: SMEs require standardized and user-friendly systems to enhance connectivity, communication, and transparency. Digitalizing internal workflows and implementing data-driven decision-making are essential for improving operational efficiency.
- **Dynamic adaptability and flexible adjustment**: Rapid deployment and configuration of systems to accommodate market changes and production needs are critical. Production systems must support continuous analysis and optimization of processes to maintain competitiveness^2,10^.
- **Cost control of maintenance**: Achieving high efficiency through system upgrades while controlling maintenance costs is a priority. Strategies should focus on asset reusability, shareability, and upgradability to ensure economic benefits throughout the system’s lifecycle.
- **Human-centered design**: Achieving a comprehensive and successful integration of I4.0 through a consensus culture is also a priority. As much as possible, requirements of human-machine collaboration must be met, and system complexity must be simplified to lower the information barriers for employees when applying new technologies and meeting customer needs. In order to achieve success, it is crucial to facilitate the adaptation of employees to new technologies and to encourage the participation of staff in the improvement of operational processes^2,11–13^.

To maintain their unique agility and flexibility, SMEs must establish interconnected networks for quick collaboration. Incorporating human roles in process improvement, democratized decision-making, and intelligent systems integration are essential steps^14,15^. BCT has emerged as a potential enabler of solutions to these challenges, offering decentralized and secure frameworks to enhance system adaptability and scalability^16^.

### Blockchain in industry 4.0

BCT has emerged as a transformative force in addressing the limitations of traditional manufacturing systems. Industry 4.0 emphasizes the integration of digital technologies to enhance operational efficiency, transparency, and sustainability. However, centralized architectures often fail to provide flexibility, real-time adaptability, and secure data sharing, all requirements if this integration is to take place^17^. BCT does offer them through its decentralized, immutable, and programmable architecture.

Key benefits of blockchain in Industry 4.0 include:

- **Real-time data sharing**: Blockchain’s decentralized nature ensures secure and instantaneous data exchanges among stakeholders, improving decision-making and operational coordination^17^.
- **Enhanced transparency and accountability**: Blockchain’s immutable ledger provides a reliable record of all transactions, enabling traceability and trust in complex supply chains and production networks^6,18,19^.
- **Automation through smart contracts**: Smart contracts reduce human error and enhance operational efficiency by enabling automated and self-executing agreements for tasks such as production scheduling, inventory management, and quality control^20^. In addition, BCT bridges a manufacturing shop floor with its Metaverse or virtual factory simulacrum, thereby creating a more immersive and trustworthy virtual environment^21^.
- **Sustainability and circular economy support**: Blockchain enables precise tracking of resources and materials, promoting efficient utilization and waste reduction, which are critical for achieving circular economy goals^6^.

However, challenges remain in deploying blockchain effectively in manufacturing. Issues such as transaction latency, scalability, and energy consumption must be addressed to ensure its widespread adoption. Emerging solutions, including sharding, off-chain processing, and optimized consensus mechanisms, are actively being developed to mitigate its limitations^7^. Furthermore, the integration of blockchain with existing manufacturing systems requires careful planning and phased deployment to minimize disruptions.

### Blockchain as digital thread

BCT enables the integration of digital threads, connecting the entire lifecycle of a product—from design and production to end-of-life management, ensuring seamless information flow across all stages and applications of a product’s lifecycle^22,23^. BCT enhances the implementation of this framework with its inherent properties of transparency, immutability, and interoperability. These features provide a trustworthy backbone for digital thread architectures, enabling intelligent manufacturing and creating new opportunities for process optimization and industrial innovation.

#### Related work

Recent studies have explored a role for blockchain unifying digital and physical systems in smart manufacturing. For instance, Lee et al.^24^ and Barenji et al.^25^ presented CPS-based architectures where blockchain enhances system collaboration and autonomous interactions. To support manufacturing customization and secure access, Perez et al.^26^ and Zhai et al.^27^ developed blockchain-integrated data architectures and access control mechanisms. These frameworks ensured traceability and customized production workflows. For industrial data scalability, Lim and Nam^28^ and Ding et al.^29,30^ demonstrated blockchain-driven MES implementations combining big data integration and smart contract logic to enable efficient production control. Customer-centric approaches have also emerged. Ding et al.^30^ and Perez et al.^26^ emphasized blockchain’s role in aligning product design and production plans with real-time customer requirements. Complementing these efforts, Lee and Su^31^ introduced a knowledge-centric framework for semantic interoperability, enriching the features blockchain provides as a digital thread backbone.

#### Literature analysis

The Industry 4.0 Reference Architecture Model (RAMI 4.0) provides a structured framework for analyzing blockchain applications across three dimensions: Life Cycle Value Stream, Hierarchy Levels, and System Integration Layers. The results are listed in Table 1. Key findings of this report are that initially, blockchain was used to address transparency in supply chains, but its use has since expanded to production planning, control, and lifecycle security. Applications have progressed from enterprise-level systems to factory and shop floor levels, with an increasing focus on real-time data synchronization and process adaptability. Furthermore, blockchain supports innovative models for asset management and process optimization through distributed ledgers and smart contracts, enabling transformative business practices.

**Table 1:** BCT frameworks for manufacturing applications.

Aspects	Elements	Ding 2021^30^	Lim 2021^28^	Barenji 2021^32^	Barenji 2022^33^	Zhai 2022^27^	Ramakurthi 2024^34^	Leng 2024^35^	Liu 2024^36^	Xiao 2024^37^	This study
Product life cycle	Customer requirement	○		●	●		Δ		○		○
	Product design			●		Δ				●	
	Production planning	●	●	○	○		●	●	●	●	●
	Production control	●	○				●	○	Δ	○	●
	Product delivery						Δ			Δ	
Hierarchy level	Enterprise and SC		Δ	Δ	Δ		Δ		○	Δ	Δ
	Factory	○	●				●	●	●	●	○
	Shop floor	●	●				Δ	●	Δ	●	●
	Station	●	●				Δ	●	Δ	●	●
	Controller	●				○		●			●
	Simple device										
System integration	Connection	○		Δ		●		○		Δ	○
	Communication	●	●	●	●	●	●	●	●	●	●
	Information	●	●	●	○	○	●	●	●	●	●
	Functional	○	Δ	○	Δ	Δ	○	●	○	○	○
	Business						Δ		○		Δ
Functional capability	Dynamic workflow updates	○	○	Δ	Δ	Δ	○	○	○	Δ	●
	Engineering change order support		Δ				Δ		Δ	Δ	●
	Real-time adaptation	○	●	○	○	●	Δ	●	○		●

● indicates full coverage or support; ○ indicates partial coverage or support; Δ indicates topics addressed but not the research focus.

In the area of Life Cycle Value Stream developments, the primary focus of early blockchain architectures on supply chain transparency was for the benefit of stakeholders during product delivery. The subsequent expansion of BCT’s role into production planning and control enabled advanced data sharing and lifecycle security. However, attention to lifecycles on the shop floor remains limited, particularly in the area of iterative upgrades and real-time adaptability.

In Hierarchy Level developments, blockchain applications have evolved from enterprise-level systems to factory floors, workshops, and individual workstations, reflecting the growing trend toward real-time collaboration and transparent management across multiple levels. These advancements facilitate seamless communication between enterprise systems and shop floor operations, bridging the gap between high-level strategic planning and on-site execution. Despite this progress, addressing the unique challenges of shop floor-level operations, such as real-time data synchronization and dynamic process adjustments, remains a key area where further research is required.

System Integration Layer developments have highlighted blockchain’s ability to enable reliable information exchange and secure functions, fostering the development of innovative business models. Research has demonstrated how blockchain can support asset management modules and optimize production systems through distributed ledgers and smart contracts. However, integrating blockchain with physical assets on shop floors continues to be a critical task for advancing these innovations.

Supplementing this RAMI 4.0-based analysis, we evaluate representative blockchain frameworks based on three features or functions essential to shop floor control: Dynamic workflow updates, ECO support, and/or real-time adaptation. Dynamic workflow updates as an issue remain underdeveloped in most studies. These studies suggest some potential for process flexibility but lack mechanisms for or evidence of achieving seamless runtime modifications. Xiao et al.^37^ focuses on lifecycle traceability without addressing workflow-level changes. ECO support is mentioned conceptually in some cases but concrete implementations or validated use cases are lacking, highlighting the difficulty of in-process modification. In contrast, real-time adaptation is an issue more explicitly addressed. Some work emphasizes real-time data integration and system responsiveness, while other offers only partial or localized capabilities. Overall, current blockchain-based systems show these early efforts at flexibility fall short in supporting live workflow updates and ECO integration. Our approach fills this gap by enabling consensus-based, contract-driven workflow adaptation with minimal disruption to ongoing operations.

Recent advances in smart contract engineering further highlight the importance of modularity and reuse. Górski introduced the AdapT framework^38^ which supports processing multiple transaction types via abstract contract components and configurable verification logic. Park et al. proposed a Smart Contract Broker^39^ to enhance contract discoverability and reuse through metadata tagging and agent-based mediation. Meanwhile, Ibba et al. developed MindTheDApp^40^ a structural analysis toolchain for Ethereum DApps that captures complex inter-contract dependencies. These approaches collectively reflect a growing focus on smart contract adaptability, traceability, and maintainability.

While blockchain demonstrates strong potential for enabling digital threads, practical limitations persist, particularly in adapting to dynamic shop floor requirements. Scalability remains a concern as high-frequency transactions challenge current frameworks. Real-time ability to handle large incremental changes in manufacturing processes requires further empirical validation. These insights underscore the need for continued research and system refinement to fully leverage blockchain’s potential with digital threads for Industry 4.0.

### Blockchain on the shop floor

BCT has significant potential for enhancing shop floor capabilities through improved data transparency, security, and automation. The automatic execution of smart contracts can be regarded as the introduction of an innovative distributed expert system that automates logistics and production management tasks, minimizing reliance on manual intervention and reducing operational error^20^. Immutable records of production step-by-step enable comprehensive traceability, fostering trust among stakeholders and allowing real-time monitoring of production processes^22,41^. Furthermore, the integration of robust encryption mechanisms ensures secure data communication across machines and operators, in particular, preventing internal cybersecurity threats on shop floors^42^.

Despite these benefits, shop floors, the core site of real-time manufacturing operations, present unique challenges that conventional blockchain frameworks struggle to address. Key requirements include high transaction volumes, low latency, and data synchronization across distributed systems. First, high transaction volumes generate significant blockchain synchronization congestion, leading to increased latency as node count and payload size grow. Second, while exact throughput and latency requirements may vary with production scale, the need for processing transactions within seconds is a baseline expectation in real-time industrial control and has been treated as a design premise throughout this study. Traditional consensus mechanisms, while secure, often fail to deliver the rapid transaction validation required for real-time operations^8,43^. Similarly, ensuring rapid data synchronization across nodes remains a formidable challenge due to stringent requirements for availability and consistency in manufacturing workflows. Ensuring consistent transaction throughput takes place in the range of thousands of transactions per second (TPS) is critical for production control. These constraints highlight the well-documented trade-offs between system performance and security, which remain a critical focus in distributed system research.

Emerging solutions such as sharding and partial replica storage offer promising avenues to address these scalability challenges. Sharding splits the blockchain into smaller, manageable parts, allowing parallel processing of transactions and reducing network congestion^7^. Partial replica storage, on the other hand, minimizes storage requirements by enabling nodes to maintain only subsets of the blockchain, improving responsiveness without compromising critical data access^44,45^. While these solutions have demonstrated potential in theoretical models and controlled simulations, their direct application to shop floor scenarios requires further empirical testing to validate claims to their feasibility and effectiveness in real-time operations.

These challenges and potential solutions are broadly understood within the blockchain research community, underscoring empirical testing’s relevance not only to manufacturing but to distributed systems in general. Acknowledging the shared nature of these challenges, a focus shift toward tailoring these technologies to the specific requirements of shop floor environments is one that the distributed systems research community can support. Future research should prioritize pilot implementations of these technologies in manufacturing settings, where their performance can be rigorously evaluated under practical conditions. This approach will provide valuable insights into their operational viability and scalability, paving the way for their broader adoption in Industry 4.0.

### Hyperledger fabric

Hyperledger fabric (HLF), a widely adopted enterprise-grade blockchain framework, offers high transaction speed, modular architecture, and robust scalability, making it particularly advantageous for shop floor operations and SME applications. Also, HLF is widely recognized for its ability to meet the demands of complex industrial applications through enhanced security, privacy, and performance optimization. HLF has a well-developed ecosystem, including reference architecture^28,46–48^ performance testing^8,49,50^ performance tuning^51,52^ and security assessment^42,47^ making it particularly advantageous for SME applications. Therefore, it was chosen to implement the BSFCS in this study.

Important features of HLF that are relevant to this study include:

1. **Ledger**: An HLF ledger contains digital assets in key-state format. After a transaction is verified, the world state of the asset is updated. The user can read the latest verified value from the world state. Transaction history is stored in an immutable record.
2. **Nodes**: HLF provides peer nodes and orderer nodes. Peer nodes can act as clients, endorsers, or submitters in the transaction process. Orderer nodes validate transactions and generate blocks.
3. **Endorsement strategy**: When a proposed transaction is sent from a peer node as a client to the HLF ledger, the endorser first simulates the transaction and returns the result to the client. Then, the client and endorser broadcast the transaction to an orderer node, which validates it and generates a transaction block. Finally, the submitter updates the ledger with the block.
4. **Smart contracts**: Smart contracts are programmatic logic defining the HLF network’s transaction rules. Users can invoke smart contracts to manipulate the ledger. Therefore, smart contracts must be well-designed to prevent users from improper actions.
5. **Chaincode**: Smart contracts can be packaged into executable programs, known as chaincodes, and then deployed to the HLF network. Chaincodes are part of the ledger, so developers can commit a new chaincode to upgrade smart contracts.
6. **Channels**: Channels are used to manage chaincodes, which enables transaction privacy. The data transmitted within a channel is only accessible to the participant nodes. A node can participate in multiple channels.
7. **Network**: The network comprises several nodes and at least one channel. It describes the physical relationship between the ledger, nodes, and channels.
8. **Membership service provider (MSP)**: An MSP manages the identities of network members and verifies their roles and permissions.

These features collectively enable HLF to address critical challenges posed by shop floor operations, such as real-time control, secure data management, and scalable transaction processing. If it can effectively leverage HLF’s modular and scalable architecture, it is hypothesized that the BSFCS will meet the unique demands of shop floor environments, delivering real-time production control, secure data exchange, and operational efficiency essential for Industry 4.0.

### Factors hindering blockchain adoption

Despite its transformative potential, blockchain technology faces persistent barriers to adoption^20^. Technical complexity, resource constraints, and managerial skepticism often deter enterprises from exploring its benefits. The lack of real-world demonstrations of its merit, in particular, stands out as a significant hurdle, preventing decision-makers from appreciating its practical value.

Demonstration projects would play a critical role in showcasing blockchain’s potential to address operational challenges such as scalability, real-time control, and secure data management. For SMEs contemplating Industry 4.0 development, such cases could provide clear evidence of how blockchain can improve efficiency and adaptability while integrating seamlessly into existing systems. They would also offer measurable outcomes, fostering confidence among stakeholders and mitigating uncertainty about the use of the technology’s feasibility.

This study highlights the urgency of implementing industry-specific pilot projects to validate blockchain’s utility. By focusing on tangible, real-world applications, these initiatives can bridge the gap between theoretical promise and operational reality, facilitating the integration of blockchain technology into the manufacturing industry.

### Gap and objectives

Despite the growing interest in blockchain technology (BCT) for Industry 4.0, current research largely neglects the specific challenges of SMEs’ shop floor operations. Current solutions predominantly address supply chains and enterprise systems but fall short in providing dynamic adaptability and phased deployment tailored to SMEs. First, existing studies lack detailed explanations or mechanisms to ensure the flexibility needed for continuous adaptation and may fail to deliver agile, real-time responses essential for shop floor operations. Second, there is an absence of incremental deployment approaches, leaving SMEs without cost-effective, phased strategies for digital transformation. Third, practical challenges specific to shop floors, such as high transaction frequency, low latency, and real-time data synchronization, remain inadequately addressed. Lastly, limited empirical validation of blockchain’s feasibility in realistic manufacturing shop floor environments heightens skepticism among SMEs, hindering broader adoption.

This study aims to address these gaps with a blockchain-based solution specifically designed for SMEs transitioning to Industry 4.0. The proposed instruction is not intended as a universal solution but as a tailored response to the operational needs of SMEs, particularly in shop floor contexts. It focuses on bridging critical gaps by enhancing dynamic adaptability and workflow management. The incremental, phased deployment approach ensures alignment with SMEs’ constrained resources and unique operational requirements. Importantly, the study emphasizes practical validation within realistic operational environments, showcasing how blockchain technology can meet specific industrial demands without disrupting existing workflows.

By combining agile methodologies with blockchain technology, this study seeks to show the way to the incremental digitization and automation of shop floor processes. The goal is not only operational flexibility but also the introduction of a verifiable and adaptable framework that can evolve alongside SME requirements. This approach ensures a gradual yet impactful process of transformation, facilitating a seamless integration of Industry 4.0 systems that respects the practical limitations and expectations of SMEs.

## Methodology

### Architecture design

**Fig. 1:**
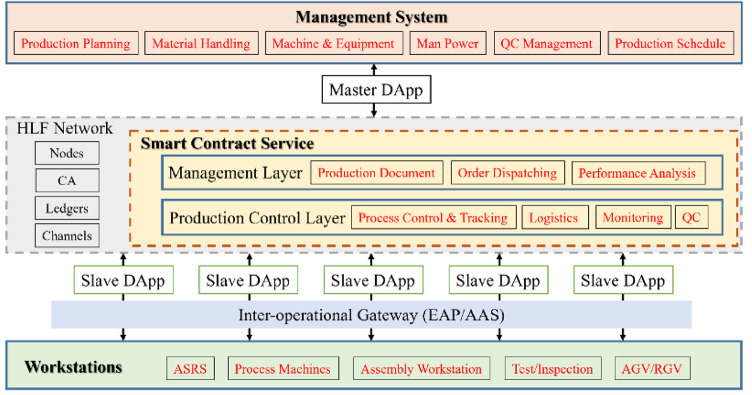
Conceptual architecture of the BSFCS.

To explore the feasibility of CWCI, a blockchain-based architecture for shop floor control is proposed. Figure 1 illustrates the top-level structure of the BSFCS. It includes a smart contract service (SCS) on an HLF network and DApps for communication with participants. The BSFCS satisfies the requirements of participants and, at the same time, regulates their behaviors. The following explains the design.

#### Participants and DApps

The main participants in the BSFCS are a manager system (MS) and workstations (WS), which need to be properly connected to the SCS. MS controls management data in the form of documents, sheets, or metadata, such as production control, equipment configuration, and material logistics. Enterprise Resource Planning (ERP) is a type of MS. WS accepts commands and monitoring from the manufacturing system to execute production processes. Participants interact with SCS through gateways known as “Decentralized Applications (DApps),” which are represented as agents between the participants and the SCS.

**Fig. 2:**
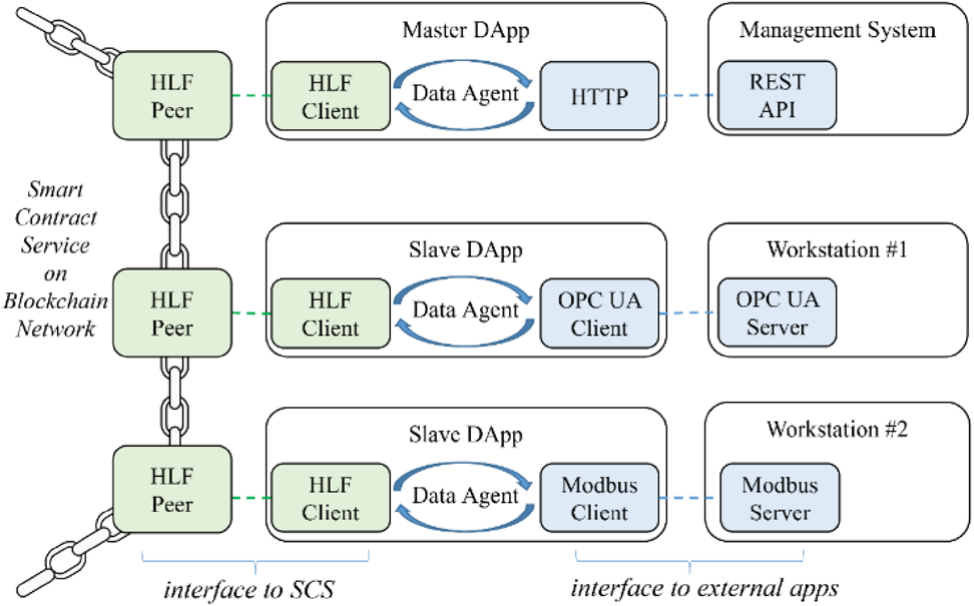
Design of DApp.

Given the situation of SMEs, the architecture and design of DApps should be implemented in ways that are easy to understand and apply^53^. A clear division of features, simple data flows, and easily extendable interaction interfaces help implementation and maintenance. Adhering to this principle and following the structure of Fig. 2, a DApp could be composed of three modules: (1) An interface for communication with participants, implemented as usual with standard communication protocols. (2) An interface that connects to the SCS service through the Fabric Gateway Client API with managed identity authentication and connections to the HLF network, allowing the DApp to invoke SCS methods. (3) A data agent that controls and converts transaction messages between participants and SCS. This design architecture is more economical and simplifies implementation and maintenance.

The most appropriate method of data exchange between MS and SCS is through Web APIs based on the HTTP protocol because it is an implementation method widely accepted by IT engineers, and this facilitates ongoing maintenance and customization. To support integration across heterogeneous data sources, a common semantic model is essential. Recent developments, such as Lee and Su’s large knowledge modeling framework^31^ and Choi and Jeong’s domain-specific Retrieval-Augmented Generation (RAG) approach^54^ align with our architecture and provide a flexible foundation for future semantic interoperability through ontology integration and context-aware metadata. While this paper does not focus on semantic modeling, the architecture is designed to support such extensions.

In contrast to MS, the integration work required for WS is more complex. To pursue efficiency and functionality, SMEs typically have a variety of machines and other equipment. This leads to the need to implement different communication protocols or connection methods during the system integration process, thereby increasing system integration’s complexity and costs. There are two ways to set up connections between WS and SCS. The first approach is to build an Equipment Automation Process (EAP) as a DApp between WS and SCS, specifically to handle production control messages. However, EAP represents a form of tightly coupled integration that introduces a certain degree of impedance to the implementation of future changes. The other is to represent the WS as an Asset Administration Shell (AAS), forming a universal I4.0 component^55,56^ that includes support for blockchain services^37^. That makes the WS a part of the AAS to form an advanced DApp, which provides a comprehensive solution, but the initialization cost may be an obstacle for SMEs. Nevertheless, the standardization of the information model and the loosely coupled design contribute to a reduction in the cost of continuing integration of WS with SCS in the future.

#### Smart contract service and authentication

As the core of the digital thread, the SCS is designed to facilitate the integration of various application systems, acting as participants, into the BSFCS via the DApp. The HLF channel effectively manages the interactions between these systems and participants by enabling secure and permissioned communication. It facilitates efficient distribution of collected data and ensures privacy through isolated channels where only authorized participants can access the relevant information. Therefore, the HLF channel plays an important role in realizing the digital thread within BSFCS.

In BSFCS, the collected data and the users are associated with several channels depending on their attributes. These channels can be extended to address data integration issues as they arise. In this study, the channels for data and users involved in control of the shop floor can be categorized into two: a management channel and a production control channel. The management channel handles production configuration settings and order management, and provides consolidated information to the production control layer. The production control channel is responsible for controlling the execution of production work by WS on the shop floor and recording the execution status and history.

SCS is also designed to manage identity authentication and to establish the permissions of DApps that are agents of participants. Authentications are assigned according to the interactions that are necessary between the participants and the channels. On the shop floor, MS can configure settings and provide information within the management channel and then send commands to the production control channel. WS follow the instructions from the production control channel and report their status.

Therefore, two types of identity authentication are defined. Master identity authentication allows a DApp to configure SCS through the management channel and obtain information about the current situation on the shop floor from the production control channel. Slave identity authentication DApps can read and update status information within the production control channel. The work of these DApps extends activity on the production control channel by controlling WS tasks strictly according to instructions defined in work orders (see below) and updating work status within the production control channel on their basis.

Only explicitly permitted interactions are allowed for security reasons. For example, for reasons of information isolation and permission limitation, slave DApps by design are not allowed to access the management channel. Also, the Master DApp does not perform direct data aggregation or consistency enforcement. These responsibilities are handled exclusively by the smart contract logic within SCS, ensuring that all transactions are validated before being committed to the ledger.

As SMEs evolve, SCS can expand to include new channels, or interaction networks can be added at the edge. As an enterprise continues to move toward I4.0, it is expected more authentication types will be introduced to enable a greater variety of intelligent applications to interact with SCS.

### Operational consensus

#### Establishment of operational consensus

Operational consensus refers to the collective decision-making within an organization concerning production processes, methods, and collaborative approaches. It aims to address challenges such as external competition, internal optimization needs, and sustainability. Achieved through collaboration and democratic participation, this consensus ensures flexibility and practical applicability while adapting dynamically to organizational goals and environmental changes.

According to the Industrial Internet Reference Architecture (IIRA) guidelines^57^, the establishment of operational consensus can be divided into the following steps: Problem Identification and Goal Setting, Functional Planning and Integration, and Feasibility Evaluation. The establishment should also follow a systematic evaluation process, including, for example, tabulating participant requirements and corresponding solutions (see Table 2) to ensure alignment with both participant and customer needs. Concepts derived from organizational management theory^58^ such as stakeholder coordination and dynamic capabilities, should be referred to while doing this. While organizational management theory provides a conceptual backdrop, its detailed application falls beyond the scope of this paper. In summary, this approach reflects principles from collaborative decision-making frameworks, which emphasize shared information, goal alignment, and adaptive mechanisms to achieve operational objectives.

**Table 2:** Operational consensus: challenges, participant requirements, and corresponding solutions.

Participant	Challenges	Requirements	Proposed solutions
Management System	Lack of coordinated oversight for sales orders and work plans, leading to inefficiencies in execution Inefficient order and material tracking systems causing delays in production planning and control Difficulty in analyzing production performance and equipment efficiency due to fragmented data sources Limited flexibility in modifying management data without causing disruptions	Deliver sales orders and product work plans for coordinated execution Manage workstations on the shop floor, handle order and material management, and provide product work planning and material lists (BOM) Obtain production history and register overall equipment efficiency (OEE) to enhance decision quality and understanding of production performance Add, update, and delete management data	Develop a smart contract module to process and synchronize sales orders and work plans in real time Implement a dynamic configuration system to update workstations and material data seamlessly Integrate data tracking mechanisms with blockchain for transparent production history and OEE analysis Enable secure, role-based access to modify management data through decentralized applications (DApps).
Workstation	Limited automation in advancing production tasks, causing delays and errors Inconsistent process conditions leading to quality variability Lack of reliable identity verification, leading to unauthorized network access or errors in workstation roles Limited traceability of production state transitions, hindering accountability	Execute production work to advance the production process Perform a process with the given conditions Provide identity and status information to access the network under system control Report the start and end points of production states	Deploy work order-specific commands through smart contracts for real-time execution of production tasks Include parameterized functions in the smart contracts to define and control production conditions Authenticate and authorize workstations through secure identity verification protocols within the blockchain network Use blockchain’s immutable ledger to record production state transitions and timestamps for traceability and accountability

#### Consensus-based workflow change integration

**Fig. 3:**
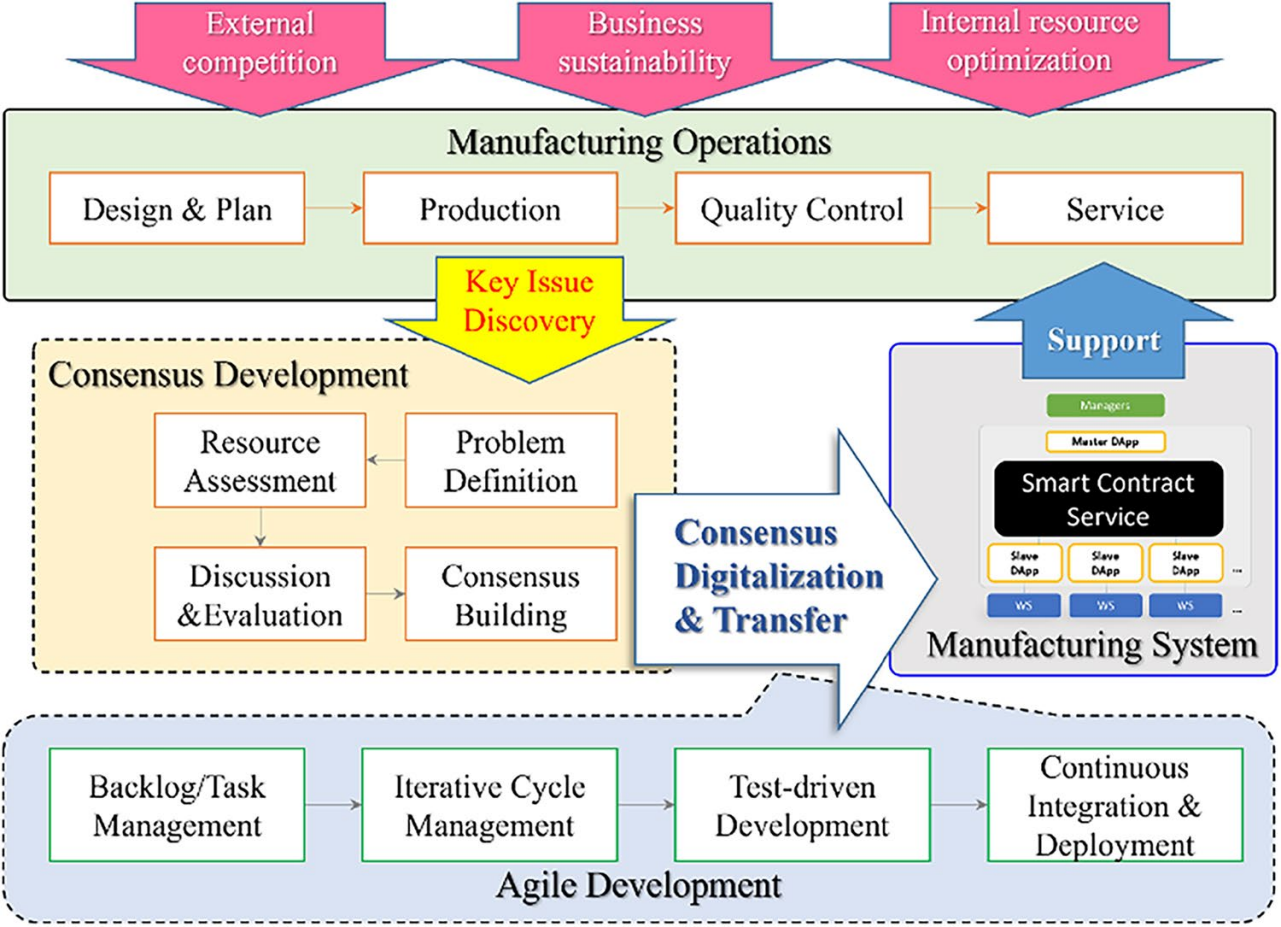
CWCI deployment paradigm.

Consensus-based Workflow Change Integration (CWCI) is the process of integrating well-defined operational consensus into an existing manufacturing system, as shown in Fig. 3. Inspired by agile project management principles, CWCI uses iterative and incremental approaches to ensure flexibility and adaptability during integration. This method delivers functional components rapidly, gathers stakeholder feedback, and continuously refines the system. It minimizes risks, supports rapid troubleshooting, and reduces errors while maintaining cost efficiency.

The successful realization of CWCI relies on the clarity and precision of the operational consensus established earlier, ensuring smooth translation into executable contract logic. In this study, smart contracts are identified as the optimal platform for implementing CWCI. Smart contracts automate operational rules, ensuring consistent execution and reducing human error. Their immutability and transparency provide a secure framework for traceability and accountability, essential in manufacturing processes. Additionally, smart contracts’ dynamic adaptability aligns closely with CWCI’s iterative approach, allowing updates to operational needs with minimal disruption.

To ensure reliable CWCI implementation, the study recommends three key practices:

1. Verification and Testing: When digitizing operational consensus into smart contracts, prioritize verifying functionality, security, and efficiency. Frequent testing cycles, akin to agile project management, help validate alignment with operational requirements and responsiveness to changes.
2. Iterative Development: Use iterative testing to confirm each implementation phase meets objectives while maintaining system performance and stability. This process synchronizes smart contract updates with evolving operational demands, ensuring trouble-free integration.
3. Well-designed Smart Contract: The iterative and incremental approach outlined in CWCI aligns closely with the modular and decentralized principles of SCS design, ensuring smooth integration and scalability in dynamic manufacturing environments. The implementation of CWCI is closely tied to the SCS, discussed in “SCS design strategy”.

By adopting agile principles such as iterative development, incremental deployment, and continuous feedback, CWCI facilitates the smooth integration of workflow changes into blockchain-based digital contracts. This adaptability enables systems to respond effectively to changing business needs. To implement CWCI successfully, the subsequent section explores SCS design strategies that provide the necessary modularity and scalability. These strategies build upon the foundational concepts of CWCI, demonstrating how modular smart contract design can enhance adaptability, ensure efficient integration, and support seamless system evolution. The discussion below assumes an implementation with Hyperledger Fabric (HLF), given that it is the best framework for CWCI because of its superior transaction performance, scalability, and robust community ecosystem.

### SCS design strategy

Transaction verification plays a crucial role in ensuring data consistency. This verification is performed at the blockchain node level through contract-defined validation logic, which includes identity checks, data structure constraints, and enforcement of process rules. The endorsement process in Hyperledger Fabric automatically rejects transactions that fail to meet these conditions, preventing them from being written to the ledger. This guarantees that only valid and trusted information becomes part of the ledger.

Additionally, the SCS forms the core layer for implementing the CWCI mechanism. This section elaborates on SCS design principles, focusing on its modularity, scalability, and transaction optimization strategies to support real-time CWCI. Key issues include transaction verification, performance optimization, data structure design, and CAP theorem considerations to address implementation challenges.

The verification mechanism is essential to maintaining data consistency and preventing downstream conflict. It eliminates the need for centralized reconciliation or post-hoc correction and ensures that all participating DApps operate only on validated, conflict-free data.

#### Transaction verification strategy

Transaction verification in blockchain-based manufacturing can adopt either on-chain or off-chain methods. While on-chain verification ensures security and transparency, off-chain verification offers flexibility and lower costs. This study recommends a phased approach: SMEs can start with off-chain verification to validate concepts quickly and transition to on-chain verification as systems stabilize to enhance trust and data integrity.

#### Performance optimization principles

This study adopts three key optimization principles to enhance system responsiveness and scalability in real-time shop floor environments. First, *on-demand committing* minimizes redundant ledger writes by submitting only necessary state updates, improving resource efficiency. Second, *transaction quality optimization* reduces payload size by eliminating low-value or duplicate data, thus preserving bandwidth and throughput. Third, *off-chain verification and caching* uses event-based scheduling and local state storage to lower computational overhead and enhance responsiveness—an approach shown to be effective in high-concurrency environments^9,59^. These strategies address the challenges of high-frequency, real-time environments, ensuring that SCS achieves efficient and scalable system performance while maintaining the integrity of manufacturing operations.

#### Efficient data design for real-time production control

**Fig. 4:**
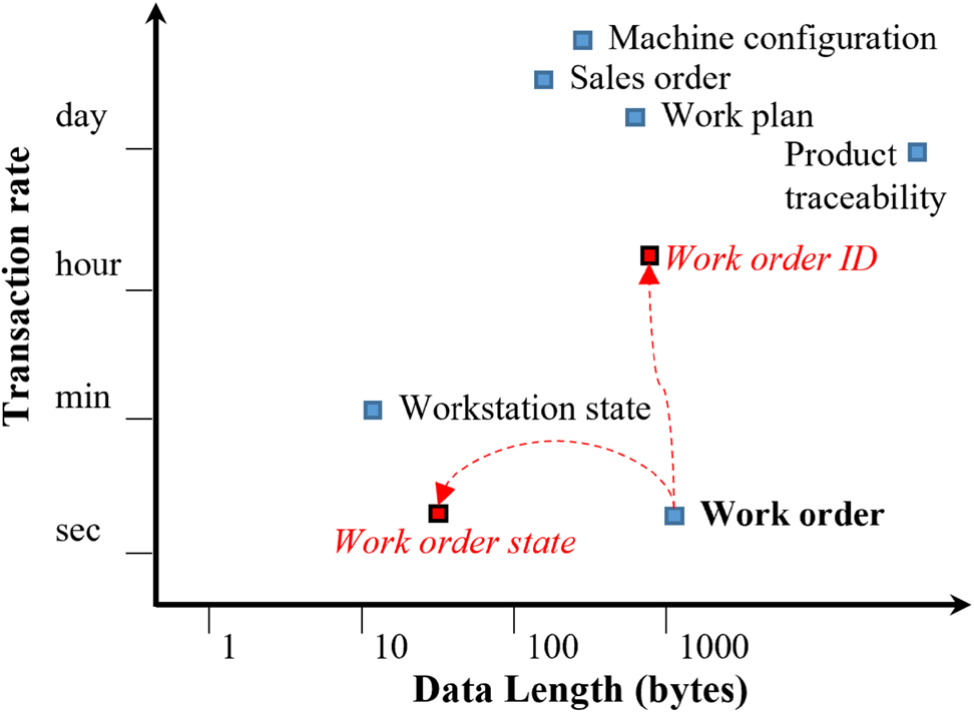
Characteristics of transaction data.

To support real-time responsiveness and scalable control, transaction data needs to be categorized into two types: management data, which changes infrequently and relates to planning or resource configuration, and control data, which updates frequently and reflects real-time production status. This difference in type is illustrated in Fig. 4.

Each record in this study, whether of management or control data, is structured with two attribute types. *Identification attributes* (e.g., order ID, routing number) remain stable and facilitate indexing and traceability. *State attributes* (e.g., current step or machine status) are frequently updated, reflecting real-time system behavior, and are designed to be lightweight to avoid performance bottlenecks and ensure real-time system responsiveness. For example, with control data when tracking a work-in-progress (WIP) item, identification attributes specify the assigned work order and routing path, while the state attribute dynamically records the WIP’s current transition step. This identification–state separation is consistently applied at both the attribute and document levels, where document identities and dynamic states are modeled as separate ledger entries with explicit lifecycle binding. This design reinforces data normalization, semantic clarity, and extensibility across all smart contract components.

This attribute separation aligns with the system’s need for both high-frequency updates and long-term consistency. Key benefits include:

- Simplified data organization via stable indexing.
- Enhanced consistency and access security through reference data isolation.
- Improved transaction efficiency using lightweight, high-frequency state updates.

By adopting this structured approach, the blockchain-based SCS can effectively manage real-time, high-frequency production data, ensuring system scalability, responsiveness, and reliability in manufacturing environments.

#### CAP theorem considerations for shared Ledger data

To manage distributed systems trade-offs and optimize ledger data sharing, the CAP theorem was applied. This theorem^60^ posits that a system can only fully achieve two out of three properties: *Consistency* (C), *Availability* (A), and *Partition tolerance* (P). In manufacturing, different data types prioritize different trade-offs. For *management data*, the study adopted a CP strategy—ensuring consistent and reliable planning data replication across nodes, even under partial network failure. For *production control data*, a CA approach was used to guarantee immediate availability and response for shop floor operations, accepting potential partition limitations. This categorization allowed the SCS to balance long-term planning reliability with real-time control performance—both essential requirements of scalable manufacturing systems.

#### Architectural design patterns in SCS

From an architectural design perspective, the proposed SCS incorporates several established design patterns to ensure modularity and scalability. The Strategy pattern governs the execution logic of different work plans. The State pattern models the progress of work-in-process items through dynamically generated Work Terms (WTs). The Command pattern structures production control, with MGMT as the invoker, smart contracts as command definitions, and machines as receivers. The interaction between DApps and external systems follows an Adapter-like structure, while the smart contract interfaces adopt the Facade pattern. The functional separation between MGMT and PROD contracts reflects the principle of Separation of Concerns. In addition, our smart contract strategy aligns with Górski’s reusable framework^38^ which advocates abstract-layer reuse and configuration-based verification. Similar to AdapT’s abstract transaction processing, our design decouples state changes and enables rule-based dynamic execution. This supports modular adaptation without compromising traceability. Further details on implementation are provided in the next section.

## System implementation

### Test environment

The proposed manufacturing system was implemented using Festo CP-Factory workstations to validate system feasibility under realistic shop floor conditions. This platform closely replicates real-world operations, making it ideal for demonstrating the practical applicability of the system. Conducted under real production conditions, the validation tested the enhanced reliability and reduced implementation barriers of the BSFCS in a realization of the benefits of theoretical blockchain technology (BCT) in practical industrial adoption.

**Fig. 5:**
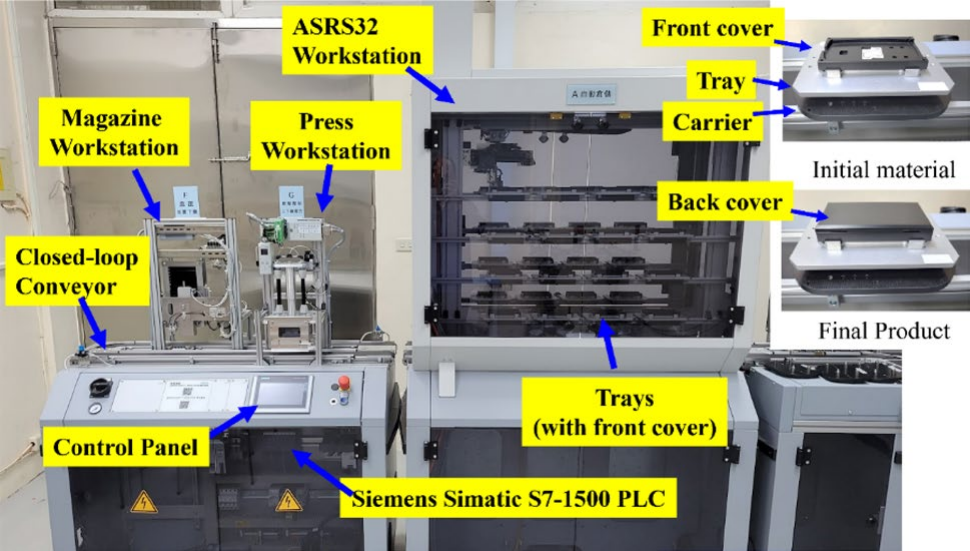
Test environment using FESTO CP-Factory.

Figure 5 shows the ASRS32, Magazine, and Press workstations connected by a closed-loop conveyor that moved product carriers with RFID tags. The factory assembled box-shaped products by sequentially placing back covers onto front covers and pressing them firmly together. The ASRS32, an automated storage and retrieval system, managed trays with front covers or completed products. The Magazine machine added back covers, and the Press machine used a pneumatic unit to secure the covers together. Each workstation operated with a Siemens Simatic S7-1500 PLC controller, accessible via an OPC UA server for data and behavior control.

To validate the BSFCS prototype, it was installed in place of the default FESTO MES4 software, and all other factory configurations were retained. The laptop on which the MES4 software and BSFCS prototype were deployed was an ASUS UX5400 with an Intel Core i7-1165G7 processor, 16GB memory, 512GB SSD, and Windows 11 Home Edition, connected to the CP-Factory via Ethernet.

### Implementation

#### Architecture

**Fig. 6:**
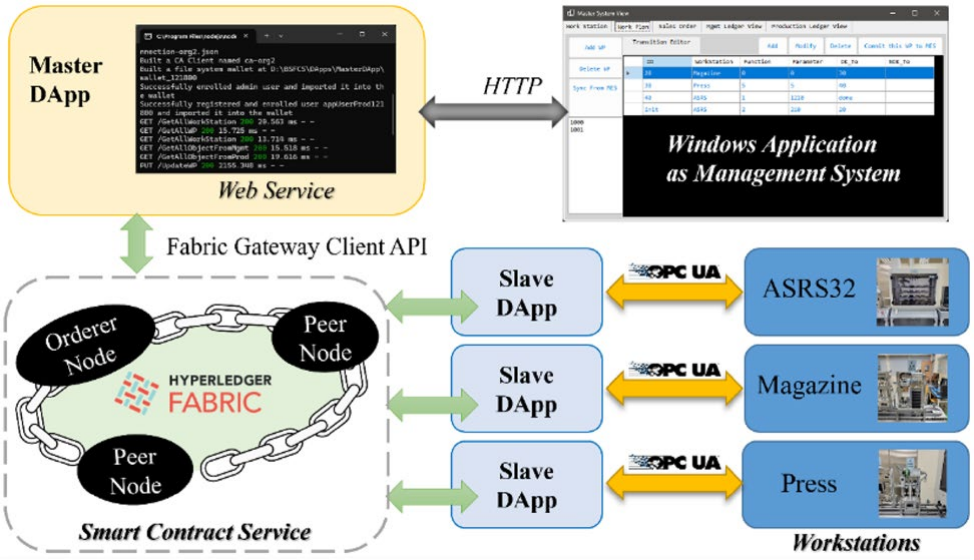
The realized architecture of BSFCS prototype.

The realized BSFCS architecture is illustrated in Fig. 6, with the SCS as its core component. SCS executed smart contracts written in JavaScript to automate production tasks. Two types of DApps were developed for external interactions: a Master DApp and Slave DApps.

- Master DApp: Developed with Node.js, utilizing the Fabric Gateway Client API (v. 2.2.20) and Express.js (v. 4.19.1) to manage interactions with SCS and MS. MS applications, such as ERP, were implemented as Windows applications using C# to interface with the Master DApp via Web APIs.
- Slave DApps: Built with Node.js and NodeOPCUA SDK (v. 2.123), handled communication with OPC UA servers on workstations, ensuring problem-free control over production equipment.

Access permissions distinguished the Master DApp and Slave DApps, providing for secure communication within the network. The interaction logic implemented in the DApps reflects the Adapter pattern, where heterogeneous external systems (e.g., ERP, OPC-UA devices) are connected to a unified blockchain interface. Additionally, the DApps act as stateful intermediaries that manage execution context and behavior, aligning with the Agent pattern.

#### Network

**Fig. 7:**
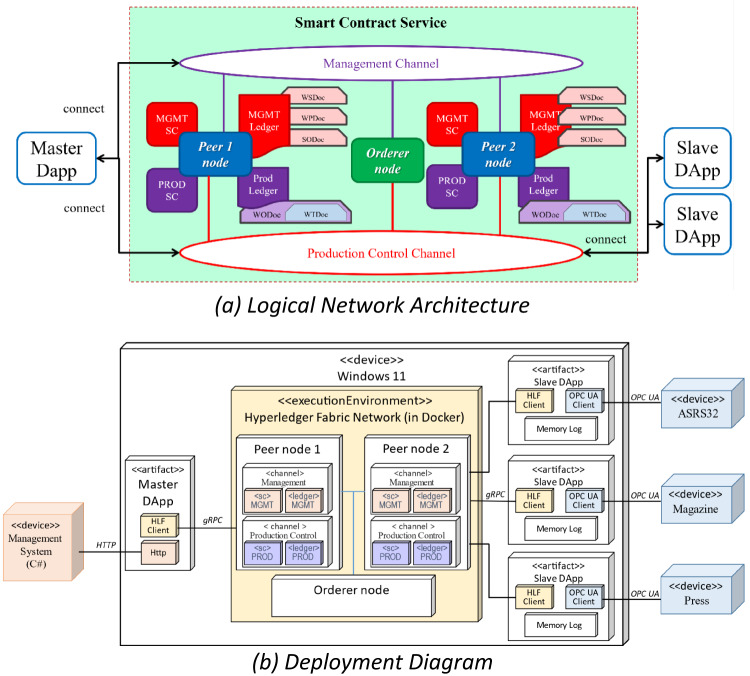
Details implementation of the proposed BSFCS.

Figure 7a illustrates the logical network architecture of the system, focusing on the abstract arrangement of smart contracts, communication channels, and participant roles. The implemented HLF network consisted of two peer nodes, one orderer node, and two channels:

- The Management Channel (MC) contained an MGMT smart contract and its ledger and handled the configuration of workstations, work plans, and sales orders.
- A PROD smart contract and its ledger were placed in the Production Control Channel (PC) to control manufacturing processes, enabling dynamic workflow execution.

The Master DApp invoked both smart contracts, while the Slave DApps were restricted to invoking the PROD contract, ensuring role-specific access to maintain system security and efficiency.

Figure 7b presents the physical and runtime distribution of system components. The system ran on a Windows 11 host and used Docker to virtualize the HLF network. The Master DApp communicated with the management system via HTTP and interacted with the blockchain via gRPC through the HLF client. Each Slave DApp included an HLF client and OPC UA client to interact with both the blockchain and on-site devices (ASRS32, Magazine, Press). The Memory Log module recorded state information locally for consistency and recovery. According to deployment needs, HLF nodes and DApps could also be distributed across multiple physical devices.

#### Design of smart contracts

The MGMT and PROD smart contracts served the distinct purposes of production configuration and control. This separation of contract roles embodied the principle of Separation of Concerns. MGMT was responsible for configuration and system setup, while PROD focused on executing and tracking production. The modular division allowed for independent development and clearer security boundaries. The smart contract interfaces were designed following the Facade pattern, exposing only essential functions to external systems and abstracting away internal complexity. This approach, combined with interface-based invocation by DApps, aligned with the Dependency Inversion Principle (DIP), ensuring loose coupling between the application layers. Their design is described below.

- Production Configuration and Management.

The MGMT smart contract enabled MS to manage the following critical BSFCS information about WS, WP, and SO:

1. Workstation:
  Captured WS manufacturing characteristics to verify WP feasibility.
  Configurable fields included WS identifiers, endpoints, protocols, and executable functions with parameters.
  Allowed dynamic adjustments to WS settings as needed.
2. Work plan:
  Defined the complete manufacturing process for each product.
  Included an identifier and transition sequence specifying the required WS, executable functions, parameters, and subsequent steps.
3. Sales order:
  Registered customer pre-orders linked to corresponding WPs.
  Contained essential fields like order identifiers, status, sales items, and conditions.

The MGMT smart contract supported operations for adding, querying, updating, and deleting configuration data. Safety mechanisms established by user consensus ensured secure and authorized access to this information.

(b) Production Tracking and Control.

Once the configuration was complete, MS could initiate the production process for SOs. The PROD smart contract generated a work order (WO) for each sales item to manage and track product progress, ensuring compliance with WP requirements.

1. Work Order Management:
  Tracked product progress and verified WP compliance.
  Updated start and end times of transitions in real-time.
2. Production Status Monitoring:
  MS could query execution progress and SO status through SCS.
  WS could validate production elements and report process statuses.

**Fig. 8:**
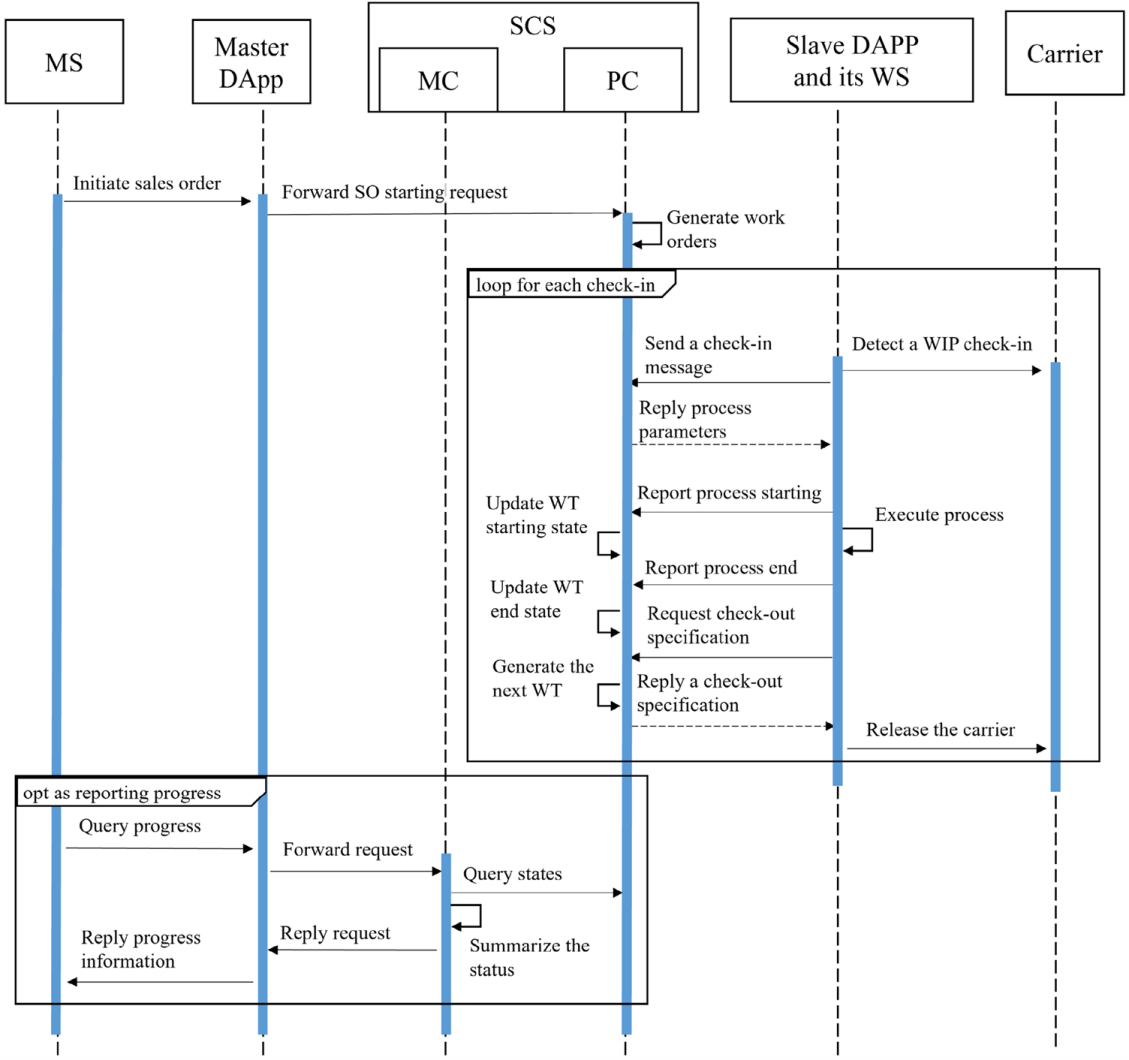
Sequence diagram of the production control process defined in SCS.

Figure 8 illustrates the interaction relationships for production control, showcasing how MS and WS operate through the SCS interface.

(c) Ledger design.

**Fig. 9:**
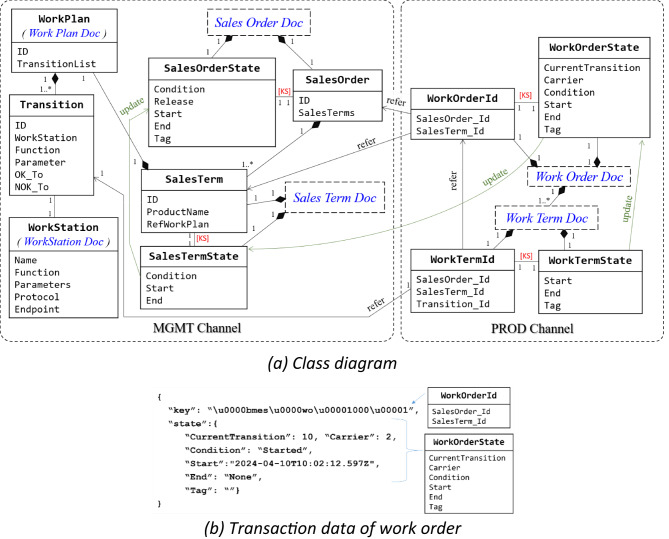
Documents in the SCS. Core documents and their states are modeled as separate ledger entries and labeled [KS] to indicate 1:1 logical linkage. Neutral associations reflect runtime lifecycle binding, and explicit cardinalities (e.g., 1:1, 1:1.*) clarify multiplicity between documents, transitions, and task elements.

The SCS ledger structure was organized around three core documents: WO, WP, and SO, each combining static identifiers, called documents, with dynamic state values to support traceability and modularity, as shown in Fig. 9a. This class diagram adopts neutral associations to reflect the system’s runtime behavior: documents and states are stored independently in the ledger, but their lifecycles are logically coupled through smart contract rules. For example, deleting a SalesOrderDoc also removes its SalesOrderState from the world state, while historical records remain traceable on-chain. This key–state pattern, labeled as [KS], is consistently applied and clarified in the figure caption.

Furthermore, the initial WO design tracked progress using a growing checklist of state entries, but this resulted in large payloads and degraded performance. To address this problem, we introduced WTs—a data-level sharding mechanism in which each WT represented a single workflow transition. This redesign ensured fixed-length, on-demand updates (typically < 20 bytes), as illustrated in the WO structure in Fig. 9b.

The runtime logic of WorkTerms is encapsulated in two smart contract functions, CheckIn and CheckOut. The CheckIn function is used to bind a free carrier to an unstarted WorkOrder or verify the conditions for the transition execution. After the transition is complete, the CheckOut function triggers the next WorkTerms or finalizes the WorkOrder. To ensure correct execution, each WT is dynamically bound to a specific workstation at runtime. In other words, the SCS verifies this binding by checking that the invoking Slave DApp and the provided workstation ID match the WorkPlan. This task–device association is enforced through smart contract logic without relying on static structural links. Algorithm 1 outlines the pseudocode of these procedures, with full implementation available in our open-source repository^61^.

**Algorithm 1:**
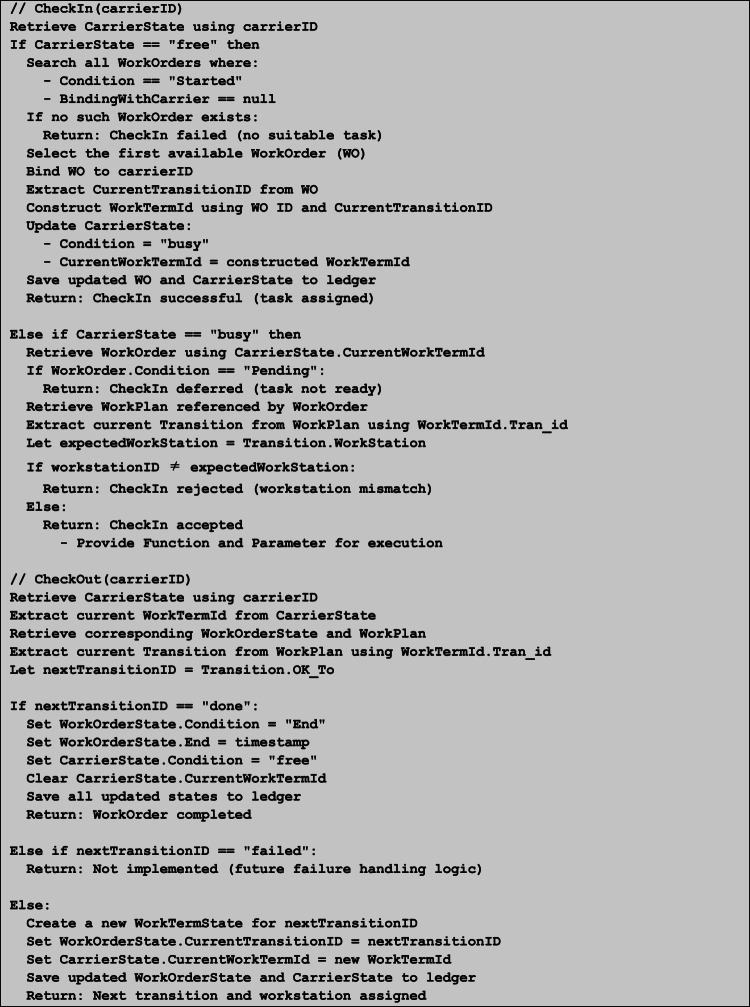
CheckIn and CheckOut Procedures for WorkTerm Transitions.

This structure significantly reduced payload size, improved transaction throughput, and enabled fine-grained monitoring of in-progress work. Its modularity supported future workflow extensions without modifying existing logic, ensuring traceability, adaptability, and scalability in shop floor operations. A complete﻿ implementation﻿ of these smart contracts, including carrier state management, event handling, and work term orchestration, is freely available in the referenced GitHub repository^61^. This paper is based on release version 1.1.0.

SCS’s optimized structure offered significant advantages. By isolating individual transitions, the data structure reduced the data payload represented by a change of all WO state information in the blockchain, resulting in storage optimization and reduced transmission for real-time tracking. The design provided granular control by enabling precise monitoring at the transition level while dynamically generating WT Docs only when needed. This on-demand committing principle minimized data overhead, improving transaction quality and system responsiveness. The SCS modularity leveraged by this optimization shows the ease with which seamless integration of new document types or workflows can be achieved. WO Doc consolidation created a detailed and immutable record of production activities and captured real-time variation even for identical product types. The robust data structure enhanced traceability, supported compliance, and aligned with Industry 4.0 objectives by ensuring transparency and adaptability in production management.

SCS’s transformative design also improves scalability, allowing SMEs to integrate evolving workflows without overhauling their systems. SCS ensures long-term relevance and efficient production management by reducing data overhead and enabling dynamic adaptations. The system supports runtime workflow changes under strict constraints. Once a WorkTerm is executed, it becomes immutable. Only future steps can be modified, and parameter updates are allowed if permitted by process logic. This mechanism applies broadly across products, though its use may be limited by external factors such as certification or quality assurance. In addition, each transaction structure may include an optional “tag” field, designed to support semantic annotation or context-specific metadata. While the current deployment does not utilize this field for semantic modeling, the tag attribute has been implemented in selected classes and is available for downstream systems to leverage when needed.

## Testing

### Validating CWCI approaches

Two simulations of production at an enterprise moving towards I4.0 were carried out to validate the study’s approach and test the feasibility of the proposed system and methods. In each case, the goals of CWCI are described, and the implementation and results are explained below.

#### Case I: initial deployment

The first case sought to demonstrate an initial BSFCS deployment in the operation of the previously described experimental factory. The goal was to test the feasibility of passing control of basic functions within the factory to the BSFCS. Specific goals included production configuration, setting up production plans, and opening sales orders. After initiating a sales order, the manufacturing system instructed the workstations to complete production tasks in sequence and to record all processes.

**Fig. 10:**
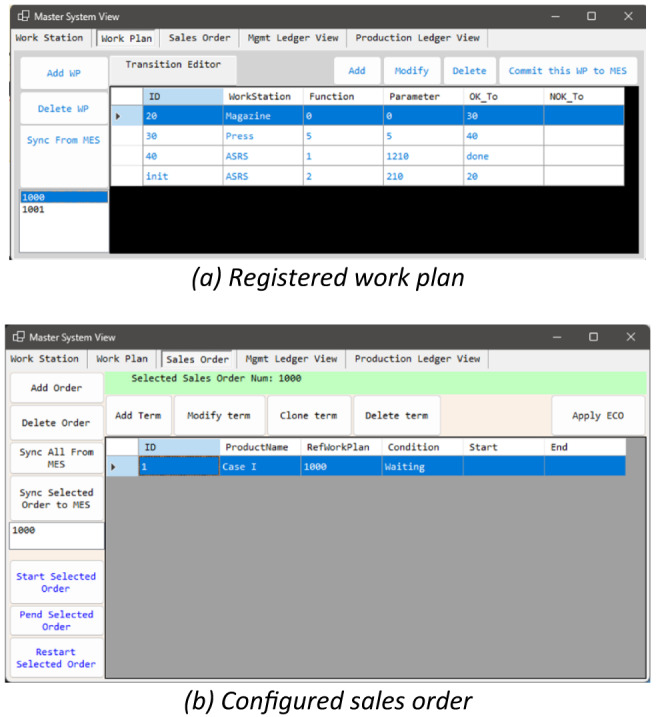
Production configuration in Case I.

After configuring the workstations, the work plan for each product included the following transitions: (1) Releasing a tray with a front cover from the ASRS32 workstation, (2) Adding a back cover to the front cover in the Magazine workstation, (3) Compressing both covers with the assigned pressure of 25 N for 5 s in the Press workstation. (4) Storing the completed product back in the ASRS32 workstation. The work plan was registered with a Windows Forms application that serves as the management system, as shown in Fig. 10a. Then, a sales order to produce the product was initiated using the Windows Forms application, as shown in Fig. 10b. It was expected that after the order was initiated, the BSFCS could instruct the workstations to complete the production tasks.

**Fig. 11:**
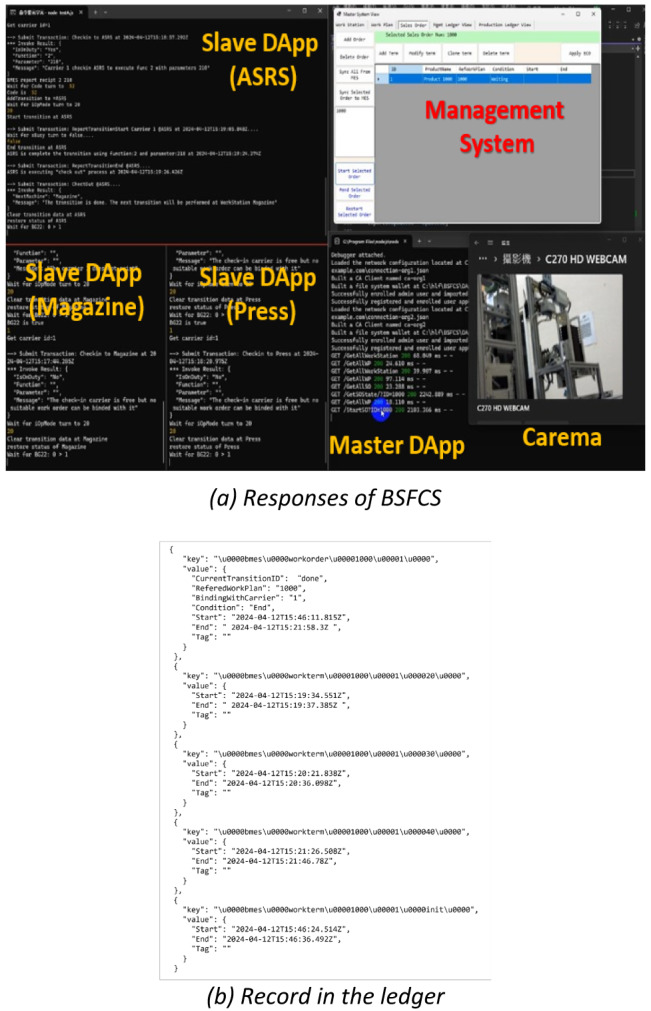
BSFCS operation with DApp responses.

Figure 11a is a screenshot of the operation in Case I, showing the DApp responses. The BSFCS controlled production, causing the workstations to complete the production tasks in sequence. After querying the ledger records, it was clear that details of all processes had been recorded, as shown in Fig. 11b. The results demonstrate the feasibility of the proposed system’s management of a production process in a workshop. The performance of the deployed BSFCS did not differ from that of the default MES software, MES4.

#### Case II: engineering order changes

This case sought to demonstrate whether deploying the BSFCS would allow SMEs to accept customers’ requests for immediate changes to orders without great problem. The specific customer request is for a change in the transition of “compressing both covers” in the Case I work plan. To improve the assembly yield rate, the pressure parameter for the two covers is to be increased from 25 N to 35 N. In this case, the SME receives the ECO from the customer during the production of the ordered item. This usually means that changes to the work plan for the sales items need to be made immediately. While satisfying customer requests is important, ECOs can be quite disastrous for SMEs. They significantly increase their production costs and are prone to introducing errors. Therefore, the goal of this case is to test whether BSFCS can assist enterprises in responding to customer needs more flexibly and efficiently.

**Fig. 12:**
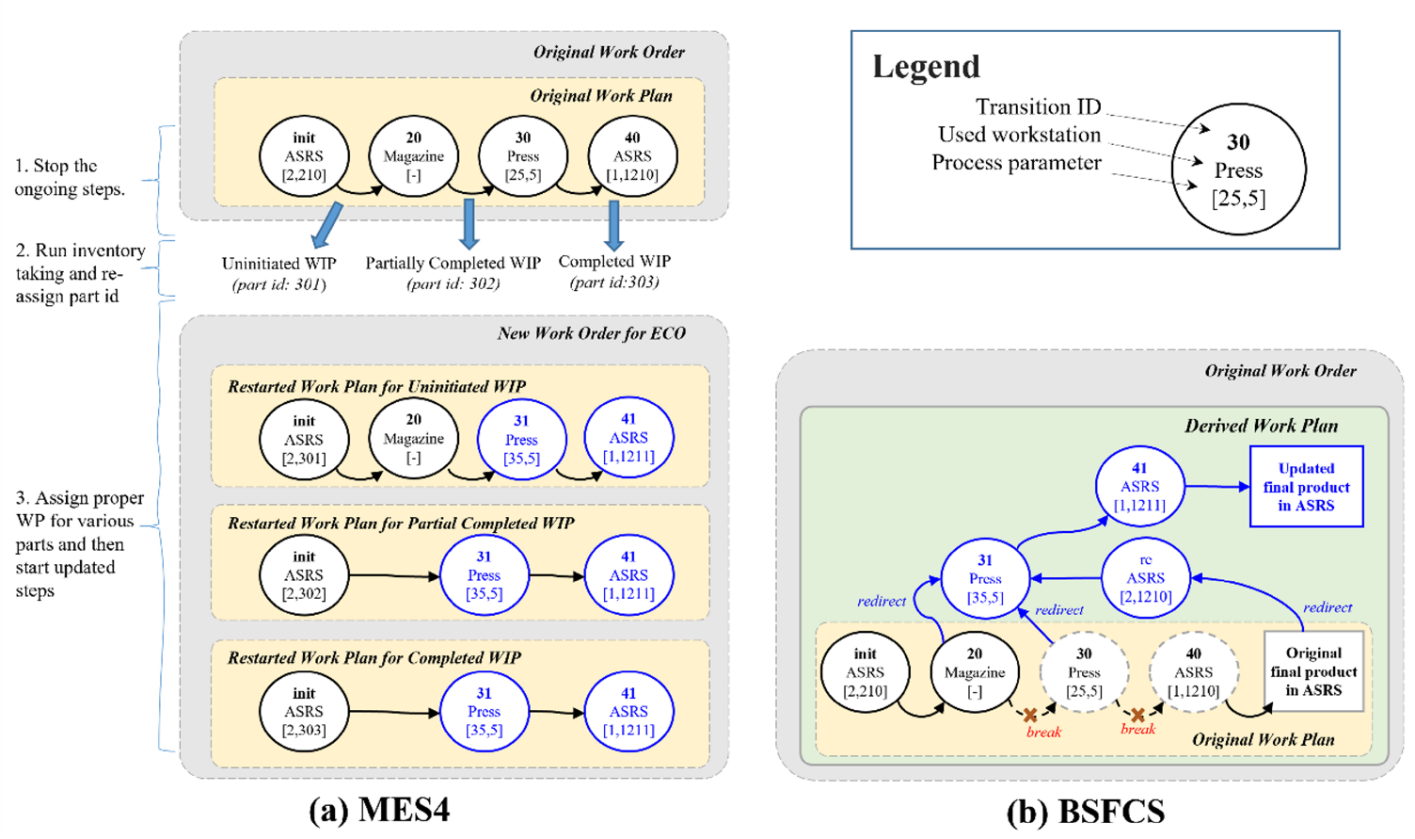
Adjustment of work plan of Case II.

First, the ECO was run with the standard MES4 control system. The usual reliability-based practice is to first stop the ongoing manufacturing process and return all WIPs to an inventory area, like the ASRS workstation. Then, through inventory taking, the status of each WIP is obtained. Finally, these statuses are used to reopen and/or initiate new processes and continue production. The operations on the WOs are presented in Fig. 12a. It can be seen that this approach is reliable but not efficient. It is usually a compromise due to the manufacturing system’s inflexibility.

In contrast, it is the author’s belief that direct adjustment of the manufacturing process in the course of production is more efficient on condition that all information about the change is reliably recorded by the manufacturing system. This concept was accepted as the new operational consensus, which was then integrated into the BSFCS. Analyzing the existing manufacturing processes, an innovative work plan change process to efficiently deal with the operational consensus can be implemented. Benefiting from the BSFCS’s design flexibility, the next WT is only generated as the corresponding transition is required. Therefore, it is expected that the production process of online WIP will be redirected to the correct plan when improvements are made to the originally planned process. Thus, a derived work plan can be followed without returning all WIP to inventory. The improvement of the derived work plan is shown in Fig. 12b.

This approach to realizing the planning change includes several steps:

1. Commit a new work plan according to the proposed work plan change process.
2. Suspend the production process within the order.
3. Change the work plan of the product within the order to the new work plan.
4. Announce the ECO to SCS. Then, all WT will be generated based on the improved work plan.
5. Continue production of the order.

This CWCI is then transformed into a digital smart contract and updated in SCS, upgrading the manufacturing system’s functionality. A test result in which there is a smooth realization of the production process change demonstrates that BSFCS adaptation is well-suited to meeting the changing requirements of SME strategy.

This case illustrates a forward-only workflow update in response to an ECO, preserving completed steps and applying new parameters only to future transitions. This approach aligns with blockchain immutability and practical manufacturing limits, though regulated steps may require additional consideration.

### Performance test

To further determine the applicability of the proposed design principles to actual production processes, a series of detailed SCS tests were conducted, focusing on its read-write performance. These tests were carried out under a testing environment identical to that described in “Test environment”. Prior to conducting the tests, it was observed that when the payload size exceeded 100 kB, the transaction failure rate increased significantly. This implies that even basic transactions may fail if the payload is too large. Based on this finding, the payloads of all performance tests were limited to a range between a maximum of 100 kB and the minimum of 10 bytes required by state updates. To ensure the representativeness and reliability of test results, stratified sampling was employed to generate the payload lengths. This method allowed the random selection of different ranges to cover overall conditions uniformly.

During testing, multiple clients were configured to asynchronously send a total of 1000 transaction requests to SCS. This setup aimed to simulate the complex scenarios encountered in real-world situations where multiple participants interact with SCS simultaneously. Four different client combination quantities were tested: 10, 25, 50, and 100 clients. In each test, a series of operations was first executed using POST requests to write data into SCS. Subsequently, GET requests were made to retrieve stored data from the ledger within SCS, thereby validating the efficiency of the write and read operations. Additionally, this process helped evaluate the system’s stability and performance when handling a large number of concurrent requests in a short period.

**Fig. 13:**
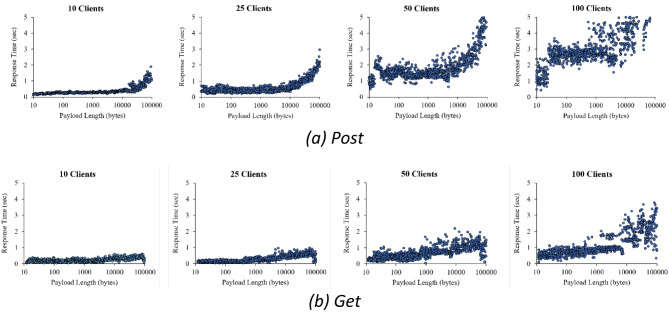
Relationship diagrams of payload and response.

Several key features of the results can be seen in Fig. 13. First, under identical conditions, the performance of GET transactions significantly surpasses that of POST. Second, the size of the payload also impacts transaction performance. Specifically, when the payload exceeds ten kB, the time required for POST operations increases greatly. Lastly, as the number of clients increases, the duration of transactions lengthens, and the stability of the transaction responses deteriorates.

The outcomes of this performance testing underscore the critical role of the design principles explored in “SCS design strategy” in crafting a manufacturing system based on blockchain technology. The transaction quality imperative requires developers to minimize both the length and frequency of payloads submitted. Strategies such as off-chain verification and on-demand committing can significantly reduce transaction counts. Furthermore, segmenting data structures into identification information and state, for example, with data-level sharding of work order state, was instrumental in reducing the length of POST transactions to less than 100 bytes and stabilizing transaction performance.

## Discussion and limitations

### Agile consensus deployment and system flexibility

This study has introduced a CWCI mechanism with SCS Design Strategy, a key innovation supporting the gradual digital transformation of SMEs envisaged in Industry 4.0. By incorporating principles of agile methodology, CWCI enables the incremental digitization and automation of workflow changes through smart contracts. This mechanism ensures that SMEs can adapt to evolving production requirements with minimal disruption, maintaining system flexibility and responsiveness.

The practical feasibility and effectiveness of CWCI with SCS design strategy were validated through two case studies conducted in an experimental factory environment. In the initial deployment (Case I), the proposed system successfully digitized shop floor control through smart contracts, ensuring seamless coordination of production machines. The operational data, securely recorded on the blockchain ledger, enhanced transparency and traceability, demonstrating the system’s capability to monitor production processes and maintain data integrity. The ECO test (Case II) further showcased the flexibility of CWCI in dynamically responding to real-time production changes. Traditional systems often require the cancellation of ongoing processes and a complete reset of production workflows when modifications occur, leading to significant time loss and resource inefficiency. In contrast, the proposed system dynamically updated the work plan through CWCI, enabling in-progress WIP items to seamlessly adjust to the revised workflow. The results imply a substantial reduction in production adjustment time and resource waste in cases of significant ECOs, highlighting the value of the system’s efficient adaptation to evolving operational requirements.

While BSFCS does not aim to perform real-time operational optimization, it provides a robust framework for executing workflow changes defined through CWCI. These changes must first be structured as operational consensus, ensuring controlled and traceable integration. As demonstrated in Case II, this design supports dynamic adaptation without disrupting ongoing production. This architecture emphasizes controlled and traceable change execution over reactive optimization. While optimal resource reallocation is beyond its current scope, BSFCS provides a stable base upon which intelligent planning tools may be integrated.

The combination of agile principles, smart contract automation, and blockchain transparency positions CWCI as a robust solution for SMEs seeking to manage operational change with low risk and high adaptability. The case studies demonstrate the system’s ability to respond to production variability while maintaining process traceability and data security. By resolving challenges associated with traditional systems, such as inflexibility and single-phase upgrades, the CWCI mechanism provides a practical pathway for SMEs to achieve a gradual yet effective transition toward Industry 4.0.

### Design strategy for performance optimization

This study adopted a streamlined approach to smart contract design, focusing on optimizing performance and ensuring system responsiveness to meet the dynamic requirements of shop floor operations. One of its contributions was mapping the document identification and dynamic state information required by manufacturing processes to the existing key-state data structure of Hyperledger Fabric, leveraging HLF’s inherent capabilities for the purposes of efficient system design. By reducing the payload size of frequent updates, this lightweight structure effectively improved transaction efficiency and system scalability. Experimental results demonstrated that transaction payloads consistently kept under 100 bytes significantly enhance transaction throughput and avoid overloading system resources.

To further avoid performance degradation, the implemented smart contract services integrated three optimization strategies: reducing redundant ledger writes, minimizing low-value transaction submissions, and leveraging local data caching for periodic updates. These strategies collectively ensured that computational overhead was minimized while the integrity and availability of critical production data was maintained. The use of off-chain verification through local caching of ledger states in the DApp, as described in “Performance optimization principles”, was particularly effective at reducing redundant read operations and contributing to faster response times, especially under conditions of high concurrency. Functional tests conducted in a simulated shop floor environment validated the effectiveness of these strategies. The performance tests indicate that the SCS design strategy of the present study prevents the significant worsening of transaction latency found in previous studies, particularly under high transaction loads. This is evidence of the system’s ability to maintain responsiveness and operational efficiency in dynamic manufacturing environments.

By addressing blockchain’s inherent limitations in high-frequency, real-time environments, the optimized smart contract design ensures that our proposed BSFCS meets the stringent demands placed on modern manufacturing systems. The integration of these principles in improving system responsiveness supports SMEs in building scalable, efficient, and reliable digital transformation processes.

### BSFCS architecture: flexibility and scalability

The BSFCS architecture demonstrates a high degree of flexibility, enabling real-time production changes without halting in-progress workflows. As illustrated in the ECO case (“Agile consensus deployment and system flexibility”), the system can redirect WIP items when process parameters are updated, ensuring continuity and responsiveness.

The modular DApp structure simplifies interactions among production WS, MS, and SCS. By decoupling these components, the system reduces integration complexity and allows SMEs to implement and expand the BSFCS incrementally, while also respecting their operational constraints. Additionally, the use of separate communication channels in Hyperledger Fabric for management and control data helps isolate transaction flows and stabilize performance under different workloads.

In addition to performance and scalability, deployment-level security is also addressed. The BSFCS incorporates channel-based access control and MSP-based role authentication to ensure secure operation. This architecture enables fine-grained permission enforcement across production and management domains, helping SMEs safeguard sensitive process data while keeping administrative complexity manageable. While this study focused primarily on system feasibility and performance, automated governance tuning, certificate lifecycle management, and security auditing frameworks can be applied to further enhance deployment robustness.

These findings collectively validate the BSFCS as a flexible and scalable solution for SMEs. The modular architecture, combined with its decentralized design and efficient communication strategy, ensures that SMEs can adapt to evolving production requirements, providing them the confidence that with the BSFCS they have a robust foundation for future expansion. While further larger-scale deployment scenarios remain to be explored, the results of the scenarios studied here provide strong evidence of the system’s practicality and its capacity to address the unique challenges faced by SMEs transitioning to Industry 4.0.

### Case validation with FESTO CP-factory

The two case studies conducted in this study provide compelling evidence for the practical applicability of the BSFCS architecture, particularly in the dynamic management of production workflows. A key strength of this validation was the use of FESTO CP-Factory, an experimental manufacturing platform explicitly designed to replicate all essential elements of real-world factory operations. The highly realistic representation of industrial shop floors of the CP-Factory ensures that the system’s performance and flexibility observed in the experiments are indicative of its success in actual production environments.

In the initial deployment case (Case I), the BSFCS successfully integrated with a number of CP-Factory modules, including automated workstations and a closed-loop conveyor system, to execute and monitor predefined production workflows. The use of smart contracts enabled seamless coordination between workstations, while the blockchain ledger securely recorded all process data, for purposes of traceability, transparency and accountability across workflows. This immutable and transparent recording mechanism ensured process traceability and validates the system’s ability to replace conventional MES solutions with a more adaptable and trustworthy control architecture. However, the consensus mechanism inherent in blockchain technology may potentially lead to delays in transaction finality, particularly under high transaction volumes. Combining blockchain with edge computing or hybrid solutions may mitigate this limitation.

The ECO case (Case II) demonstrates the system’s capacity to handle dynamic production changes with minimal disruption. Through CWCI and smart contract design, BSFCS allows production processes to be reconfigured seamlessly, avoiding the typical high cost and manual intervention associated with ECOs in conventional systems. Using the CP-Factory as the experimental platform, the BSFCS efficiently updated process parameters in response to real-time operational changes, redirecting in-progress WIP items to modified workflows without requiring a reset or manual intervention. This level of adaptability highlights the system’s ability to support agile production processes—an essential requirement for SMEs operating in volatile markets.

A comparison between BSFCS and Festo MES4 is shown in Table 3. The main differences are explained as follows: The primary production control process of MES4 runs on a central computer and manages the manufacturing workflows through the EAP at each workstation. MES4 requires a higher investment in security measures and scalability infrastructure to mitigate the risks of single points of failure. In contrast, the BSFCS distributes its production control processes across nodes, effectively minimizing system-wide failures due to single-point disruptions. Leveraging its blockchain-based framework, the system offers notable advantages, including robust data security, comprehensive data traceability, and inherent system self-healing capabilities.

To further contextualize the comparison, we may highlight the differences in data update speed and client scalability. The performance of MES4 in our setup relied on a file-based Access database, which limited concurrency but still achieved approximately 0.1-second-level update speeds. Server-based MES systems (e.g., SQL Server) can further reduce latency to the millisecond level and support thousands of concurrent clients. The BSFCS, built on blockchain, achieved ~ 1–2 s update latencies and supported around 20 TPS under implemented settings. While this represents a constraint on maximum client connectivity, the results reflect the recognized trade-off required to enable higher transparency, decentralized governance, and smoother integration of workflow changes.

**Table 3:** Comparison between BSFCS and Festo MES4.

Aspect	Conventional MES (MES4)	This study (BSFCS)
System Architecture	Centralized	Decentralized, blockchain-based
Data Update Speed	Fast (File-based DB: ~0.1s; Server-based DB: sub-second or millisecond level)	Near Real-Time (Blockchain-based: typically 1–2 s due to consensus delay)
Traceability	Limited, dependent on manual reporting	Immutable, blockchain-enabled traceability
Security	Moderate, prone to single-point failures	High, distributed, and cryptographically secure
Adaptability to Changes	Rigid requires halting production	Agile supports live Engineering Change Orders (ECO)
Scalability	Limited by central processing capacity	Modular, scalable through distributed nodes
Self-Recovery	None (can be supplemented with backup systems at high cost)	Built-in fault tolerance and self-healing via consensus
Compliance and Governance	Manual updates, prone to human error	Automated, rule-based through smart contracts
Operational Transparency	Partial, dependent on central logs	Complete, transparent through distributed ledger
Integration Complexity	High, requires custom solutions	Moderate, standardized DApp architecture simplifies connections
Real-Time Monitoring	Efficient, quick updates from centralized databases supported	Limited by consensus, potential delays in node-to-node synchronization
Maximum Client Connectivity	High (hundreds to thousands of concurrent clients supported by server-based DBMS)	Moderate (limited by blockchain TPS, e.g., ~ 20 TPS in current setups)
Change Management	Complex, disruptive	Seamless, iterative through CWCI
Upgrade Cost	High, centralized upgrades required	Low, incremental updates are possible

The successful implementation on the CP-Factory platform will give SMBs confidence that the BSFCS will work effectively in real factory environments. FESTO’s CP-Factory replicates critical manufacturing elements, including automated material handling, programmable logic controllers (PLCs), and real-time monitoring systems, making it an ideal testbed for validating industrial systems. By achieving seamless control and flexible production adjustments within this environment, the BSFCS demonstrates its readiness for deployment in actual SME shop floors where similar operational challenges exist.

### Future directions: blockchain as digital thread

This study establishes the BSFCS as a foundation or framework for SME transition toward Industry 4.0. Moving forward, positioning blockchain as a digital thread provides a natural and robust pathway for the integration of emerging technologies and the enhancement of system adaptability, scalability, and intelligence. A digital thread, underpinned by blockchain provides a secure and immutable platform for unifying shop floor data across all production stages—from planning and execution to real-time monitoring and optimization. By positioning blockchain as a digital thread, this study lays the groundwork for SMEs’ integration of advanced technologies and the enabling of intelligent and data-driven manufacturing ecosystems.

The expectation that the integration of artificial intelligence (AI) and machine learning (ML) technologies into the BSFCS in the future will be trouble-free is the product of its leveraging of blockchain’s inherent strengths of transparency, traceability, and data integrity. AI-driven analytics will be able to leverage real-time blockchain data to optimize production scheduling, predict equipment failures, and adjust workflows responding to shop floor conditions dynamically. This integration will transform the BSFCS into an intelligent decision-making platform supporting predictive and adaptive production control strategies.

To support such AI integration with richer semantic understanding, future work may also explore semantic modeling across product lifecycle stages. Although the current deployment focuses on execution-level control, the system already includes a tag field in selected transaction classes, enabling downstream interpretation and annotation without altering the core ledger structure. This extensibility provides a natural entry point for integrating domain ontologies, data lakes, or LLM-based summarization approaches to unify production data with upstream (e.g., design, planning) or downstream (e.g., maintenance, service) information.

Additionally, by processing data closer to production endpoints with edge computing, a digital thread’s capacity for real-time decision-making and operational responsiveness is strengthened, particularly in time-sensitive manufacturing workflows. By distributing computational loads closer to shop floor operations, such edge computing minimizes latency in executing smart contracts and accelerates data-driven decision-making. Enhancements of this form are particularly valuable in dynamic manufacturing environments where rapid response times are critical for maintaining production efficiency.

To address the increasing scale and complexity of industrial operations with which SMEs must cope, future research could explore performance scalability within the digital thread framework. Techniques such as blockchain sharding and transaction parallelization offer promising solutions for improving throughput and reducing latency, ensuring the BSFCS can support higher transaction volumes and real-time data synchronization without compromising system performance.

In this study, a form of data-level sharding has already been implemented within smart contract logic. For instance, work order states are divided into lightweight Work Terms, which are dynamically generated and committed only when needed. This approach significantly reduces transaction payload size and enhances responsiveness, as demonstrated in our performance tests. However, this sharding strategy operates only at the application layer. Future work should investigate ledger-level sharding mechanisms, which allow multiple partitions of the blockchain network to process transactions in parallel. Such techniques could further increase throughput and scalability for large-scale shop floor environments.

In conclusion, integrating AI/ML and edge computing within a blockchain-enabled digital thread creates significant opportunities for further development of the BSFCS into an intelligent, real-time production control system. Future integration with tools such as smart contract and network analyzers^39,40^ could further support smart contract reuse and structural consistency, especially in LLM-assisted and large-scale multi-agent settings^31^. These enhancements would build on the current study’s contributions and establish a clear pathway for BSFCS evolution, underscoring its potential to serve as a transformative solution for SMEs navigating the complexities of Industry 4.0.

## Conclusion

This study developed a Blockchain-based Shop Floor Control System (BSFCS) using Hyperledger Fabric to assist small and medium-sized enterprises (SMEs) in achieving a flexible and scalable transition toward Industry 4.0. By integrating agile methodologies and smart contract service design strategy, the system effectively addresses the challenges of operational adaptability, real-time production control, and data transparency in dynamic manufacturing environments. The main contributions of this study are summarized as follows:

1. Proposing a Consensus-based Workflow Change Integration (CWCI) Mechanism: The CWCI mechanism combines agile principles with blockchain technology to facilitate the incremental digitization of workflow change. This enables SMEs to dynamically respond to production changes with minimal disruption, as demonstrated in the engineering change order (ECO) case. The CWCI’s ability to manage in-progress work-in-process (WIP) items and seamlessly adapt workflows highlights its practical advantages over traditional systems.
2. Design and Validation of the BSFCS Architecture: The proposed system features a modular, decentralized architecture that simplifies integration with existing shop floor operations. Key innovations, such as the Identification-State data structure and performance optimization strategies, ensure lightweight and efficient transaction processing. Experimental results demonstrated that state updates consistently remained under 100 bytes, supporting real-time production requirements while maintaining system responsiveness. Performance testing (see “Performance test” and Fig. 13) further showed that with payloads under 100 bytes, the BSFCS achieved an average throughput of approximately 20 transactions per second (TPS), demonstrating its responsiveness under typical shop floor conditions.
3. Practical Validation through FESTO CP-Factory: The system was rigorously tested within the FESTO CP-Factory, a platform replicating real-world manufacturing conditions. The initial deployment (Case I) successfully digitized shop floor control processes, while the ECO test (Case II) showcased the system’s ability to dynamically accommodate production changes. The CP-Factory validation provides strong evidence of the system’s feasibility and readiness for implementation in actual SME environments.
4. Enhancing Digital Thread with Blockchain: The BSFCS serves as a foundation for developing a blockchain-enabled digital thread, unifying shop floor data across the production lifecycle while ensuring data integrity, traceability, and transparency. Future extensions of the system will be able to seamlessly integrate AI/ML technologies for predictive analytics and use edge computing techniques to enhance real-time data processing, further advancing the system’s intelligence and scalability.

While the BSFCS demonstrates promise, certain limitations remain. Blockchain’s performance under high transaction volumes requires further optimization, such as through advanced techniques like sharding and transaction parallelization. Furthermore, this study primarily focuses on technological feasibility and does not address broader organizational or cultural factors impacting SMEs’ digital technological development. Future real-world implementations of the BSFCS should consider stakeholder perspectives to ensure successful adoption and alignment with enterprise goals.

In conclusion, this study presents the BSFCS as a transformative solution for SMEs, combining blockchain technology, agile methodologies, and digitalized consensus mechanisms. The system’s validated flexibility, scalability, and dynamic adaptability provide a clear pathway for SMEs to navigate the challenges of Industry 4.0. The BSFCS serves as a robust foundation for real-time production control and future technology integration, empowering SMEs to achieve sustainable growth and operational excellence in the Industry 4.0 era.

## Supplementary Information

Below is the link to the electronic supplementary material.

Supplementary Material 1

## Data Availability

The source code supporting the findings of this study is openly available at: GitHub: https://github.com/chintelin/BSFCS . Functional test data are not separately provided, as they can be reproduced by executing the source code. Performance test data generated during the study are available from the corresponding author upon reasonable request.
